# Effects of Glycyrrhizic Acid on Peroxisome Proliferator-Activated Receptor Gamma (PPAR*γ*), Lipoprotein Lipase (LPL), Serum Lipid and HOMA-IR in Rats

**DOI:** 10.1155/2010/530265

**Published:** 2009-11-16

**Authors:** Chia Yoke Yin, Ton So Ha, Khalid Abdul Kadir

**Affiliations:** ^1^School of Science, Monash University, Sunway Campus, 46150, Bandar Sunway, Malaysia; ^2^School of Medicine and Health Sciences, Monash University, Sunway Campus, 46150, Bandar Sunway, Malaysia

## Abstract

Studies on ligand binding potential of glycyrrhizic acid, a potential agonist to PPAR*γ*, displayed encouraging results in amelioration of metabolic syndrome. The regulation of gene cassettes by PPAR*γ* affects glucose homeostasis, lipid, lipoprotein metabolism and adipogenesis. This study was performed to determine the effects of GA on total PPAR*γ* and LPL expression levels, lipid parameters and HOMA-IR. Oral administration of 100 mg/kg GA for 24 hours resulted in an increase in insulin sensitivity with decreases in blood glucose, serum insulin and HOMA-IR. Improvement in serum lipid parameters was also observed with a decrease in triacylglycerol, total cholesterol and LDL-cholesterol and an elevation in HDL-cholesterol. GA administration also resulted in up-regulation of total PPAR*γ* and LPL expression levels in the visceral and subcutaneous adipose tissues, abdominal and quadriceps femoris muscles, as well as liver and kidney, with a significant up-regulation only in the visceral adipose tissue, abdominal and quadriceps femoris muscles. Thus, oral administration of 100 mg/kg GA for 24 hours improved insulin sensitivity and lipid profiles and induced upregulation of total PPAR*γ* and LPL expression levels in all studied tissues.

## 1. Introduction

Metabolic syndrome, or more commonly known as syndrome X, encompasses a constellation of risk factors such as visceral (abdominal or central variant) obesity, insulin resistance (IR), glucose intolerance, dyslipidaemia and hypertension [1]. Studies have shown that obesity and insulin resistance are the two predominant underlying mechanisms for the development of metabolic syndrome [2]. Insulin resistance is also a characteristic in most patients with type 2 diabetes mellitus (T2DM) [2].

T2DM involves the impairment of insulin action and secretion from the pancreatic *β*-cells. Both factors will collectively increase blood glucose levels [3]. Research has proven that impaired insulin secretion together with peripheral insulin resistance is the main regulator behind the incidence of T2DM [4]. 

Peroxisome proliferator-activated receptor gamma (PPAR*γ*) gene is a well known susceptibility gene for T2DM due to the presence of polymorphisms within the gene. PPAR*γ* is a ligand-activated transcription factor originating from the nuclear hormone receptor superfamily [5]. PPARs form heterodimers with retinoid X receptors (RXRs) and bind to the hexanucleotidic PPAR responsive element (PPRE), thereby regulating the expression of target genes involved in lipid and carbohydrate metabolism [6]. 

Since PPAR*γ* is a major regulatory transcription factor, it is involved in the regulation of several biochemical pathways through the transcriptional control of target genes [7]. Among the pathways involved through the regulation of gene cassettes by PPAR*γ* are glucose homeostasis, lipid and lipoprotein metabolism, inflammatory and immune responses as well as adipogenesis [8].

PPAR*γ* is the master regulator of fat cell function that governs differentiation of pre-adipocyte precursor cells into mature adipocytes capable of lipid filling as well as the mediation of hormone and cytokine expression [9]. In addition to an increase in smaller and more insulin sensitive adipocytes, PPAR*γ* activation also promotes apoptosis of mature, lipid-filled visceral adipocytes and a redistribution of the free fatty acids towards the subcutaneous adipose tissues [5]. The increase in adipocyte number increases the lipid storage capacity and indirectly confers protection towards non-adipose tissues in the event of excessive lipid accumulation [10].

Lipoprotein lipase (LPL), a water-soluble enzyme, liberates free fatty acids through the hydrolysis of ester bonds of water-soluble substrates such as TAG, phospholipids and cholesterol esters [11]. LPL is synthesized and highly expressed in the adipose tissues, cardiac and skeletal muscle, kidney, and mammary glands while lower levels are present in the liver, adrenal and brain [12]. The expression of LPL is governed by the activation of PPAR*γ* by cognate ligands as LPL is a downstream gene of PPAR*γ*. The PPAR*γ*/RXR complex would bind to the PPRE present in the promoter region of the LPL gene and increases the LPL gene expression [13]. The induction of lipoprotein lipase synthesis by PPAR*γ* is mainly in the mature adipocytes in order to increase local generation of free fatty acids [14].

It has been reported that Glycyrrhizic acid (Glycyrrhizin or Glygyrrhizinate, GA), the bioactive compound extracted from roots of licorice plants, has anti-diabetic properties [15]. In view of the above, the present study was undertaken to investigate the effects of orally-administered GA on total PPAR*γ* and LPL expression levels and HOMA-IR in rats.

## 2. Materials and Methods

### 2.1. Animals Studies

Male Sprague Dawley rats (*Rattus norvegicus*) (6 weeks old) obtained from University Malaya's Animal House (University Malaya, Malaysia) were housed 1 per cage with free access to food and drinking water. They were maintained on a 12-hour light-dark cycle in a room with controlled temperature (24 ± 1°C) and humidity (55 ± 10%). The use and handling procedure of animals had been approved by the Monash University Animal Ethics Committee according to the 2004 NHMRC Australian Code of Practice for the Care and Use of Animal for Scientific Purposes and Relevant Victorian Legislation (Prevention of Cruelty to Animals Act 1986) (AEC: SOBSB/MY/2006/46).

### 2.2. In-Vivo Assay

The rats were divided into 2 groups (control and treated) [8 rats per group]. The treated group was given 100 mg kg^−1^ of GA (Sigma Chemical Co., St. Louis, MO, USA) orally while the control group was given tap water without GA. The rats were fed ad libitum with Glenn Forest stock-feeder rat chow during the treatment period of 24 hours. Upon completion of the projected treatment period, the rats fasted for 12 hours prior to humane sacrifice under the influence of anaesthesia via intraperitoneal administration of pentobarbital sodium (120 mg/kg, IP).

Blood was withdrawn from the apex of the heart for measurement of glucose and insulin levels. Tissues (subcutaneous and visceral adipose tissues, abdominal and quadriceps femoris muscles, and liver and kidney) were harvested for measurement of total PPAR*γ* and LPL expression levels.

### 2.3. Laboratory Assay

Blood glucose was analyzed by glucose oxidase method employing a Powerwave XS Microplate Scanning Spectophotometer (BIO-TEK, USA). Enzyme immunoassay (LINCO-Millipore Corp., US) was used to measure serum insulin. For the estimation of insulin sensitivity, the homeostasis model assessment of insulin resistance (HOMA-IR) was calculated (concentration of glucose × concentration of insulin/22.5) [16].

Total cholesterol and TAG were measured with a Randox CH200 Cholesterol kit (Randox, UK) and a Wako Triglyceride E kit (Wako, Japan). To determine the level of HDL-cholesterol, HDL-cholesterol was first separated from the LDL and VLDL fractions by precipitation of the latter two using the Randox CH203 HDL precipitant, followed by a cholesterol assay using the Randox CH200 Cholesterol kit. The levels of total cholesterol, TAG and HDL cholesterol obtained were used to calculate LDL-cholesterol using the Friedewald formula [17].

### 2.4. Real-Time Reverse Transcription Polymerase Chain Reaction (RT-PCR) Quantification of Total PPAR*γ* and LPL Expression

#### 2.4.1. RNA Isolation and RT-PCR

Total mRNA was obtained from subcutaneous and visceral adipose tissues, abdominal and quadriceps femoris muscles, and liver and kidney of each animal using Qiagen RNeasy Mini Kit and Qiagen RNeasy Lipid Tissue Mini Kit according to the protocol provided by the manufacturer. The concentration of the mRNA was determined by measuring the absorbance at 260 and 280 nm. The quality of the mRNA was confirmed by ethidium bromide staining of 18S and 28S ribosomal RNA after electrophoresis on 1.2% agarose gel. 1 *μ*g of total RNA was reverse-transcribed with Qiagen Omniscript reverse transcriptase (Qiagen, USA). The expression of total PPAR*γ* and LPL was assessed by quantitative RT-PCR, using Opticon Monitor 3 (MJ Research Inc, UK) with Locked Nucleic Acid (LNA) Dual-labeled Fluorogenic Probes detection. The forward and reverse primers for the amplifications of total PPAR*γ* and LPL expression levels are listed in Table 1. The comparison of total PPAR*γ* and LPL expression levels between control and treated rats was performed using the Comparative Ct (ΔΔCt) Method, with BAC as reference, treated group as target and control group as calibrator.

### 2.5. Statistical Analysis

Data were expressed as mean ± standard error of mean. Statistical analyses of total PPAR*γ* and LPL expression levels were performed using the Relative Expression Software Tool (REST©) MCS Beta 2006 while that of all other parameters was performed using the Statistical Package for the Social Sciences (SPSS) version 16.0 (SPSS Inc., Chicago, IL, USA). A *P* value of ≤.05 was considered significant.

## 3. Results

### 3.1. Biochemical Analysis

**Figure 1:**
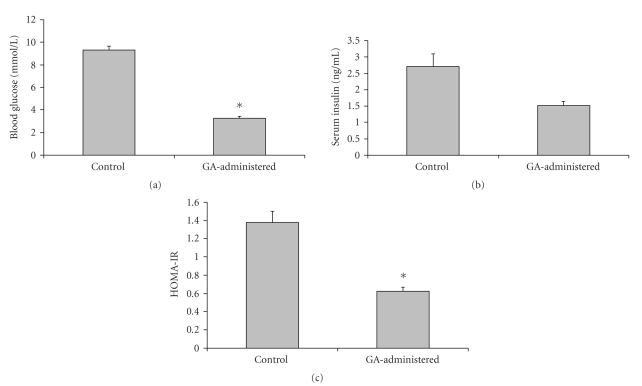
(a) Blood glucose, (b) serum insulin, and (c) HOMA-IR levels in GA-administered rats (∗ indicates *P* < .05).

Rats administered with GA showed 65% (control, 9.31 ± 0.40 mmol/L; treated, 3.26 ± 0.23 mmol/L) and 37% (control, 2.71 ± 0.41 ng/mL; treated, 1.53 ± 0.12 ng/mL) decrease in blood glucose (*P* < .05) and serum insulin (*P* > .05) levels, respectively, compared to the control rats (Figure 1). Insulin resistance, calculated as HOMA-IR, was 83% (control, 1.38 ± 0.12; treated, 0.62 ± 0.05) lower in the GA-administered rats compared to the control ones (Figure 1) (*P* < .05).

**Figure 2:**
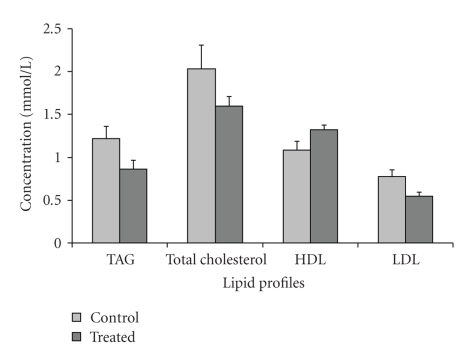
Serum lipid profiles in GA-administered rats.

Consistent improvement in all lipid parameters was observed in the GA-administered rats relative to the control (Figure 2). Mean serum TAG, total cholesterol and LDL-cholesterol showed a 28.72%, 21.55%, and 28.60% reduction, respectively, (control, 1.22 ± 0.14 mmol/L; treated, 0.87 ± 0.10 mmol/L; control, 2.04 ± 0.13 mmol/L; treated, 1.60 ± 0.11 mmol/L; control, 0.77 ± 0.08 mmol/L; treated, 0.54 ± 0.05 mmol/L, resp.) (*P* > .05). Rats administered with GA showed an elevation in HDL-cholesterol by 17.95% compared to control rats (control, 1.08 ± 0.10 mmol/L; treated, 1.32 ± 0.06 mmol/L) (*P* > .05).

### 3.2. Total PPAR*γ* and LPL Expression in Rat Tissues

**Figure 3:**
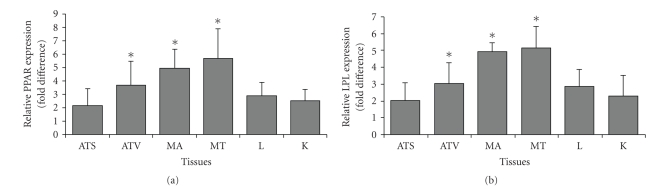
Relative expression (fold difference) for (a) total PPAR*γ* and (b) LPL in tissues with BAC as reference, GA-administered group as target, and control group as calibrator. ATS: Subcutaneous adipose tissue, ATV: Visceral adipose tissue, MA: Abdominal muscle, MT: Quadriceps femoris muscle, L: Liver, K: Kidney (∗ indicates *P* < .05).

Abdominal muscle (MA) displayed the highest fold difference expression of total PPAR*γ* followed by quadriceps femoris (MT), visceral adipose tissue (ATV), subcutaneous adipose tissue (ATS), liver (L), and kidney (K) (Figure 3). Compared with the control groups, relative expression of total PPAR*γ* in GA-administered rats was higher in all tissues. The MA, MT and ATV displayed a significant increased fold difference (*P* < .05) of 15.16 ± 5.58 fold, 13.09 ± 3.11 fold and 8.85 ± 3.62 fold, respectively, when compared to the control group. The ATS, L and K also displayed increased fold difference of 2.00 ± 0.81 fold, 5.07 ± 2.17 fold and 2.26 ± 1.08 fold, respectively, albeit an insignificant increase (*P* > .05).

The increased LPL expression in all studied tissues (Figure 3) was similar to the expression of PPAR*γ* namely the MA, MT, and ATV also displayed a significant increase in fold difference (*P* < .05) and of 11.02 ± 4.06 fold, 8.82 ± 3.01 fold, and 4.53 ± 2.66 fold, respectively. A similar trend of non-significant increase (*P* > .05) in fold difference was observed in the ATS (1.27 ± 0.85 fold), L (2.76 ± 1.97 fold) and K (2.18 ± 1.44 fold).

## 4. Discussion

The present study showed significant decrease in blood glucose and HOMA-IR in rats administered with 100 mg/kg of GA for 24 hours. Takii et al. [18] and Nakagawa et al. [19] reported that normal and obese genetically diabetic KK-A^y^ mice given ethanolic extract of licorice roots displayed suppression of blood glucose levels. Activation of PPAR*γ* was shown to cause a decrease in blood glucose levels through the inhibition of glucagon gene transcription and secretion by inhibiting the transcriptional activity of PAX6 [20].

The decrease in the serum insulin levels could be attributed to the insulin sensitization properties bestowed upon the activation of PPAR*γ*. Under a normoglycaemic state, circulatory insulin would oversee the suppression of hepatic glucose production and enhancement of glucose uptake by peripheral organs [5]. Insulin has also been implicated in the biosynthesis of LPL [21], where the insulin-signaling pathway activates PPAR*γ*, which subsequently binds to the PPRE at the LPL gene promoter region to upregulate LPL gene expression [22]. A significant drop in the serum insulin levels was found in diabetic KK-A^y^ mice for 7 weeks fed with ethanolic extract of licorice roots [18]. Results from our study exhibited a similar trend but the drop was non-significant probably due to the shorter treatment duration.

A decrease in HOMA-IR in the present study could be due to the lowered insulin and glucose levels as HOMA-IR reflects the product of glucose output and insulin secretion [23]. Therefore, a lower HOMA-IR indicates improved insulin sensitivity. Hanyu et al. [22] demonstrated that plasma LPL activity reflects whole-body insulin sensitivity and is negatively correlated with the HOMA-IR. The results of this study were in agreement with Hanyu et al. [22] where the higher insulin sensitivity (lower HOMA-IR) in the GA-administered rats developed concomitantly with the increase in tissue LPL expression. In addition, the activation of PPAR*γ* also regulates the adipocyte hormone gene expression to improve insulin sensitivity. Activation of PPAR*γ* increases the adiponectin expression which potentiates insulin sensitivity in the liver and skeletal muscles [24].

In the present study, increase in tissue LPL expression was consistent with the improvement of serum lipid parameters in the GA-administered rats. A reduction in serum TAG, total cholesterol and LDL-cholesterol together with an elevation in HDL-cholesterol was observed. The decrease in TAG in GA-administered rats may be attributed to the action of GA which causes an increase in its tissue uptake. Berthiaume et al. [25] have demonstrated that inhibition of 11*β*-hydroxysteroid dehydrogenase 1 (11*β*-HSD1) could reduce hepatic very low density lipoprotein (VLDL) which may have increased the hepatic free fatty acid oxidation. Thus, this could have caused the reduction in TAG as it is required to boost the VLDL secretion. The decrease in LDL-cholesterol in GA-administered rats may be due the lowering effect of VLDL since LDL is a derivative of VLDL. Elevation of HDL-cholesterol in GA-administered rats may be due to the increased apo A-I production since the rate of HDL synthesis is dependent on the production of apo A-I [26].

With reference to Figure 3, increased LPL expression was also seen in adipose tissue. LPL is a downstream gene that is regulated by the activation of PPAR*γ*, where an increase in the expression of PPAR*γ* would lead to an increase in the expression of LPL [27]. Thus, the increase in PPAR*γ* expression is accompanied by a similar significant increase in LPL gene expression. LPL is a key enzyme in the metabolism of triglyceride-rich lipoproteins and plays the role of a gatekeeper in energy metabolism by controlling the generation of fatty acids. In adipose tissue, the increase in LPL production could enhance the clearance of plasma triglycerides and provide the (pre-)adipocytes with additional fatty acids, which can further stimulate the transactivation capacity of PPAR [28]. The upregulation of PPAR*γ* in adipose tissues had been shown to cause an increase in glucose transporter-1 (GLUT4) and c-Cbl associating protein (CAP), pivotal both for the translocation of GLUT4 to the cell surface and for the enhanced glucose uptake [5].

Although the liver is an organ involved in glucose and lipid metabolism, only low levels of PPAR*γ* were detected in the liver [14]. Although the exact mechanism involved in the activation of PPAR*γ* in the liver remains unknown, it has been postulated that a circulating factor might be responsible for the stimulation of hepatic total PPAR*γ* transcription during a state of increased energy availability [29]. The upregulation of hepatic total PPAR*γ* would in turn activate several genes such as adipocyte fatty acid-binding protein (aP2) and fatty acid translocase (FAT/CD36), previously present only in trace amounts in the liver of lean mice. Activation of aP2 in the liver prevents the detrimental effects of free fatty acids on cells and membranes while that of FAT/CD36 facilitates fatty acid transport which ultimately lowers serum triacylglycerol levels and improves insulin sensitivity [30].

Muscles are pivotal tissues in glucose homeostasis as it is the primary organ for the insulin-stimulated glucose disposal. Significant increases were observed in total PPAR*γ* and LPL expression levels. Higher fold difference expression was found in quadriceps femoris compared to the abdominal muscle. This could be due to higher physical activity undertaken by the quadriceps femoris muscle and its higher metabolic capabilities [31]. Thus, the increase in PPAR*γ* expression in the GA-treated rats suggests possible upregulation by GA. Increase in PPAR*γ* expression correlates with enhanced insulin-stimulated glucose uptake into the muscles mediated by increased insulin-stimulated P13K activity and translocation of GLUT4 towards the cell membrane [32]. Thus, the significant increase in total PPAR*γ* expression of the quadriceps femoris muscle in the GA-treated rats was probably due to enhancement in insulin-stimulated glucose uptake mechanisms. Wang et al. [33] have reported that a lack of LPL expression in the skeletal muscle could result in insulin resistance in other metabolic tissues (e.g., liver and kidney). This could ultimately lead to obesity and systemic insulin resistance.

The expressions of PPAR*γ* and LPL in the kidneys are lower compared to those of adipose tissue, complementing the fact that the kidney is not the main regulatory organ involved in lipid and glucose homeostasis [5]. The upregulation of total PPAR*γ* in the kidney in the present study suggests the potential regulation of renal sodium and water reabsorption [34]. Most importantly, the activation of PPAR*γ* also renders renoprotective effects on the kidneys through improved glucose metabolism [35].

Both the visceral and subcutaneous adipose tissues displayed the highest expression levels of total PPAR*γ* and LPL compared to the control tissues, thus substantiating their importance in adipogenesis and adipocyte remodeling. Increased PPAR*γ* expression levels in the liver and skeletal muscles may indicate an increase in circulatory glucose uptake by glucose transporters. The PPAR*γ* expression levels were the lowest in the kidney, which plays an important role in the regulation of electrolyte concentration and blood pressure. The upregulation of total PPAR*γ* and LPL expression levels coupled with a decrease in blood glucose and serum insulin levels as well as HOMA-IR indicated improvement in insulin sensitivity most likely due to ligand-binding activation of PPAR*γ*.

In conclusion, we have demonstrated that GA lowered serum insulin, blood glucose and HOMA-IR significantly in rats compared to the control group. Improvements in serum lipid parameters with a decrease in triacylglycerol, total cholesterol and LDL-cholesterol and an elevation in HDL-cholesterol were observed. All six tissues (liver, kidney, abdominal and quadriceps femoris muscles, and visceral and subcutaneous adipose tissue) examined displayed upregulation of total PPAR*γ* and LPL expression levels. Quadriceps femoris and abdominal muscles and visceral adipose tissue showed a significant increase in total PPAR*γ* and LPL expression. Increase in expression levels of total PPAR*γ* and LPL in all the tissues was representative of their functions as regulators of glucose homeostasis.

## Figures and Tables

**Table 1:** Primer sequences used for real-time PCR.

Genes	Primers	
Total PPAR*γ*	Forward	5′-CCCTGGCAAAGCATTTGTAT-3′
	Reverse	5′-GGTGATTTGTCTGTTGTCTTTCC-3′
LPL	Forward	5′-CAGCAAGGCATACAGGTG-3′
	Reverse	5′-CGAGTCTTCAGGTACATCTTAC-3′
*β*-actin (BAC)	Forward	5′-CAGCAAGGCATACAGGTG-3′
	Reverse	5′-CGAGTCTTCAGGTACATCTTAC-3′
